# Supplementary material for: Machine learning approaches classify clinical malaria outcomes based on haematological parameters
Source: BMC Med. 2020 Nov 30;18:375. doi: 10.1186/s12916-020-01823-3 (PMC7702702; doi:10.1186/s12916-020-01823-3)

**Supplementary information**

**Machine learning approaches classify clinical malaria outcomes based on haematological parameters**

*Morang’a et al.*

**Table of Contents**

[Supplementary tables 2](#_Toc54090704)

[Table S1. The list of haematological parameters adopted from laboratory procedure manual by the CDC [52]. 2](#_Toc54090705)

[Table S4. The odds ratio of median categories providing the odd of being diagnosed with either nMI, UM, and SM. The median categories were; low and high levels. 4](#_Toc54090706)

[Table S5. Performance evaluation of six machine learning models to classify clinical malaria outcomes 5](#_Toc54090707)

[Supplementary figures 7](#_Toc54090708)

[Fig. S1. Word cloud of clinical manifestations using clinicians/doctors notes or suspected infections. 7](#_Toc54090709)

[Fig. S2. Artificial Neural Network Schematic. 8](#_Toc54090710)

[Fig. S3. Plot for the training and validation history of the ANN. 9](#_Toc54090711)

[Fig. S4. Case by case analysis of the classification capability of the ML models. 10](#_Toc54090712)

[Fig. S5. Density estimates of the haematological parameters between nMI and UM cases for sub-sampled data from Kintampo only. 11](#_Toc54090713)

[Fig. S6. Density estimates of the haematological parameters between nMI, and UM cases for sub-sampled data from Kintampo only, as well limit of children under 4 years of age. 12](#_Toc54090714)

# Supplementary tables

## Table S1. The list of haematological parameters adopted from laboratory procedure manual by the CDC [52].

| **Cell** | **Parameter** | **Measured** | **Pulse Size** | **Reported Units** |
| --- | --- | --- | --- | --- |
| WBC | **White Blood Cell Count**  This is the number of leukocytes measured directly, multiplied by the calibration constant, and expressed as n x 10^3^ cells/µL | WBC bath | ≥35 fL | n x 10^3^ cells/µL |
| RBC | **Red Blood Cell Count**  This is the number of erythrocytes measured directly, multiplied by the calibration constant, and expressed as n x 106 cells/µL | RBC bath | 36 to 360 fL | n x 106 cells/µL |
| Hb | **Hemoglobin Concentration**  Weight (mass) of hemoglobin determined from the degree of absorbance found through photocurrent transmittance is: Hb (g/dL) = Constant x log10(Reference % T / Sample % T) | WBC bath | 525 nm | g/dL |
| Hct | **Hematocrit**  This is the relative volume of packed erythrocytes to whole blood, computed as: Hct (%) = RBC × MCV/10 | Computed | RBC x MCV/10 | % Percent |
| MCV | **Mean Cell Volume**  This is the average volume of individual erythrocytes derived from the RBC histogram. | Derived from RBC histogram | # x size of RBC/ Total RBC | fL |
| MCH | **Mean Cell Hemoglobin**  This is the weight of hemoglobin in the average erythrocyte count, computed as: Hb / RBC x 10 | Computed | Hb/RBC x 10 | pg |
| MCHC | **Mean Cell Hemoglobin Concentration**  This is the average weight of hemoglobin in a measured dilution, computed as: Hb / Hct x 100 | Computed | Hb/Hct x 100 | g/dL |
| RDW | **Red Cell Distribution Width**  RDW represents the size distribution spread of the erythrocyte population derived from the RBC histogram. It is the coefficient of variation (CV), expressed in percent, of the RBC size distribution. | Derived from RBC histogram | CV expressed in % of the RBC size distribution | % Percent |
| Plt | **Platelet Count**  This is the number of thrombocytes derived from the Plt histogram and multiplied by a calibration constant. This number is expressed as: n x 103 cells/µL | RBC bath | 2 to 20 fL | n x 103 cells/µL |
| MPV | **Mean Platelet Volume**  MPV is the average volume of individual platelets derived from the Plt histogram. It represents the mean volume of the Plt population under the fitted Plt curve multiplied by a calibration constant, and expressed in femtoliters. | Derived from Plt histogram | Mean volume of Plt population under the fitted curve x constant | fL |
| NE% | **Neutrophil Percent**  The percentages of leukocytes from each category are derived from the scatterplot. | Derived from scatterplot | # cells inside NE area/# cells inside total cell area x 100 | % Percent |
| NE # | **Neutrophil Number**  The absolute numbers of leukocytes in each category are computed from the WBC count and the differential percentage parameters. | Absolute number | NE%/100 x WBC Count | 103 cells/µL |
| LY% | **Lymphocyte Percent**  The percentages of leukocytes from each category are derived from the scatterplot. | Derived from scatterplot | # cells inside LY area/# cells inside total cell area x 100 | % Percent |
| LY# | **Lymphocyte Number**  The absolute numbers of leukocytes in each category are computed from the WBC count and the differential percentage parameters. | Absolute number | Ly%/100 x WBC Count | 103 cells/µL |
| MO% | **Monocyte Percent**  The percentages of leukocytes from each category are derived from the scatterplot. | Derived from scatterplot | # cells inside MO area/# cells inside total cell area x 100 | % Percent |
| MO# | **Monocyte Number**  The absolute numbers of leukocytes in each category are computed from the WBC count and the differential percentage parameters. | Absolute number | MO%/100 x WBC Count | 103 cells/µL |

*PDW - Platelet Distribution Width and Pct - Plateletcrit are NOT for diagnostic use and do not print. Coulter uses the value for PDW is an internal check on the reported platelet parameters. The table outlines the description of the parameters, abbreviation, mode of measurement, pulse size, and the units of reporting.

## Table S4. The odds ratio of median categories providing the odd of being diagnosed with either nMI, UM, and SM. The median categories were; low and high levels.

| *Predict clinical diagnosis of UM* | *Estimate* | S.E | OR | 2.5% | 97.5% | Pr (>Chi) | |  |
| --- | --- | --- | --- | --- | --- | --- | --- | --- |
| (Intercept) | 1.73 | 0.45 | 5.62 | 2.34 | 13.49 |  | |  |
| WBC count - High Levels | -0.1 | 0.2 | 0.90 | 0.60 | 1.34 | *** | |  |
| RBC count - High Levels | -0.1 | 0.17 | 0.91 | 0.65 | 1.27 | *** | |  |
| Hb level - High Levels | -0.43 | 0.23 | 0.65 | 0.42 | 1.03 | *** | |  |
| Hematocrit - High Levels | -0.71 | 0.22 | 0.49 | 0.32 | 0.76 | *** | |  |
| Platelet count - High Levels | -2.26 | 0.14 | 0.10 | 0.08 | 0.14 | *** | |  |
| Mean cell volume - High Levels | -0.21 | 0.2 | 0.81 | 0.55 | 1.20 |  | |  |
| Lymphocytes count - High Levels | -0.77 | 0.19 | 0.46 | 0.32 | 0.67 | *** | |  |
| Mixed cells % - High Levels | -0.26 | 0.17 | 0.77 | 0.55 | 1.08 | *** | |  |
| Neutrophils % - High Levels | 0.3 | 0.36 | 1.35 | 0.68 | 2.74 | *** | |  |
| Mean cell hb conc - High Levels | 0.23 | 0.16 | 1.26 | 0.93 | 1.71 | ** | |  |
| Mean corp hb - High Levels | 0.58 | 0.21 | 1.79 | 1.18 | 2.73 | ** | |  |
| RBC Dist width % - High Levels | 0.7 | 0.16 | 2.02 | 1.48 | 2.76 | *** | |  |
| Mean platelet vl - High Levels | -0.32 | 0.15 | 0.72 | 0.54 | 0.96 | ** | |  |
| Platelet distr width - High Levels | -0.4 | 0.14 | 0.67 | 0.50 | 0.88 | ** | |  |
| Lymphocytes % - High Levels | -0.29 | 0.35 | 0.74 | 0.38 | 1.49 |  | |  |
| Mixed cells count - High Levels | -0.23 | 0.19 | 0.80 | 0.55 | 1.16 |  | |  |
| Neutrophils count - High Levels | 0.34 | 0.2 | 1.40 | 0.94 | 2.09 | . | |  |
|  |  |  |  |  |  |  | |  |
| Predict clinical diagnosis of SM | **Estimate** | **S.E** | **OR** | **2.5%** | **97.5%** | | **Pr (>Chi)** | |
| (Intercept) | 3.87 | 1.03 | 47.79 | 6.75 | 390.36 |  | |  |
| WBC count - High Levels | -0.46 | 0.59 | 0.63 | 0.20 | 2.00 | *** | |  |
| RBC count - High Levels | -2.06 | 0.57 | 0.13 | 0.04 | 0.40 | *** | |  |
| Hb level - High Levels | -2.25 | 0.69 | 0.11 | 0.03 | 0.40 | *** | |  |
| Hematocrit - High Levels | -1.31 | 0.73 | 0.27 | 0.06 | 1.10 | *** | |  |
| Platelet count - High Levels | -3.24 | 0.42 | 0.04 | 0.02 | 0.09 | *** | |  |
| Mean cell volume - High Levels | -1.33 | 0.5 | 0.26 | 0.10 | 0.68 | *** | |  |
| Lymphocytes count - High Levels | 1.57 | 0.57 | 4.83 | 1.64 | 15.33 |  | |  |
| Mixed cells % - High Levels | -0.42 | 0.45 | 0.66 | 0.27 | 1.59 | ** | |  |
| Neutrophils % - High Levels | 0.73 | 0.84 | 2.08 | 0.41 | 10.74 | ** | |  |
| Mean cell hb conc - High Levels | 3.97 | 0.54 | 52.89 | 19.25 | 161.79 | *** | |  |
| Mean corp hb - High Levels | -0.69 | 0.55 | 0.50 | 0.17 | 1.46 | . | |  |
| RBC Dist width % - High Levels | 1.55 | 0.43 | 4.72 | 2.07 | 11.10 | *** | |  |
| Mean platelet vl - High Levels | -4.52 | 0.52 | 0.01 | 0.00 | 0.03 | *** | |  |
| Platelet distr width - High Levels | 3.27 | 0.47 | 26.33 | 10.89 | 70.04 | *** | |  |
| Lymphocytes % - High Levels | -0.94 | 0.77 | 0.39 | 0.08 | 1.71 |  | |  |
| Mixed cells count - High Levels | -0.17 | 0.55 | 0.84 | 0.28 | 2.49 |  | |  |
| Neutrophils count - High Levels | 0.38 | 0.54 | 1.46 | 0.51 | 4.20 |  | |  |

***OR-odds ratio, S.E – Standard Error, Pr (>Chi) –statistical estimate***

## Table S5. Performance evaluation of six machine learning models to classify clinical malaria outcomes

|  | **ANN** | **UM vs nMI** | **SM vs nMI** |
| --- | --- | --- | --- |
|  | **Model type** | Binary model | Binary model |
| **Data splitting** |  |  |  |
|  | Total data (100%) | n=1681 | n=1504 |
|  | Training & validation data (80%) | n=1345 | n=1204 |
|  | Testing data (20%) | n=336 | n=300 |
|  | **Training performance** |  |  |
|  | Training accuracy | **0.856** | **0.985** |
| ANN | **Testing performance** |  |  |
|  | Testing accuracy | 0.801 | 0.960 |
|  | Kappa | 0.583 | 0.913 |
|  | Precision | 0.780 | 0.971 |
|  | Recall | 0.717 | 0.918 |
|  | F1- Score | 0.747 | 0.944 |
| Logistic Regression | **Training performance** |  |  |
|  | Training accuracy | 0.817 | 0.962 |
|  | **Testing performance** |  |  |
|  | Testing accuracy | 0.815 | 0.947 |
|  | Kappa | 0.615 | 0.884 |
|  | Precision | 0.803 | 0.934 |
|  | Recall | 0.734 | 0.917 |
|  | F1- Score | 0.767 | 0.925 |
| Multivariate Adaptive Regression Splines | **Training performance** |  |  |
|  | Training accuracy | 0.820 | 0.980 |
|  | **Testing performance** |  |  |
|  | Testing accuracy | 0.786 | 0.973 |
|  | Kappa | 0.542 | 0.942 |
|  | Precision | 0.742 | 0.972 |
|  | Recall | 0.685 | 0.954 |
|  | F1- Score | 0.712 | 0.963 |
| Decision Trees | **Training performance** |  |  |
|  | Training accuracy | 0.794 | 0.937 |
|  | **Testing performance** |  |  |
|  | Testing accuracy | 0.777 | 0.930 |
|  | Kappa | 0.533 | 0.848 |
|  | Precision | 0.704 | 0.899 |
|  | Recall | 0.731 | 0.907 |
|  | F1- Score | 0.717 | 0.903 |

|  | **ANN** | **UM vs nMI** | **SM vs nMI** |
| --- | --- | --- | --- |
|  | **Model type** | Binary model | Binary model |
| Random Forest | **Training performance** |  |  |
|  | Training accuracy | 0.817 | 0.982 |
|  | **Testing performance** |  |  |
|  | Testing accuracy | 0.815 | 0.960 |
|  | Kappa | 0.614 | 0.914 |
|  | Precision | 0.808 | 0.936 |
|  | Recall | 0.723 | 0.954 |
|  | F1- Score | 0.765 | 0.945 |
| SVM | **Training performance** |  |  |
|  | Training accuracy | 0.822 | 0.981 |
|  | **Testing performance** |  |  |
|  | Testing accuracy | **0.827** | **0.970** |
|  | Kappa | 0.650 | 0.936 |
|  | Precision | 0.849 | 0.955 |
|  | Recall | 0.761 | 0.996 |
|  | F1- Score | 0.803 | 0.959 |

# Supplementary figures

## Fig. S1. Word cloud of clinical manifestations using clinicians/doctors notes or suspected infections.

**
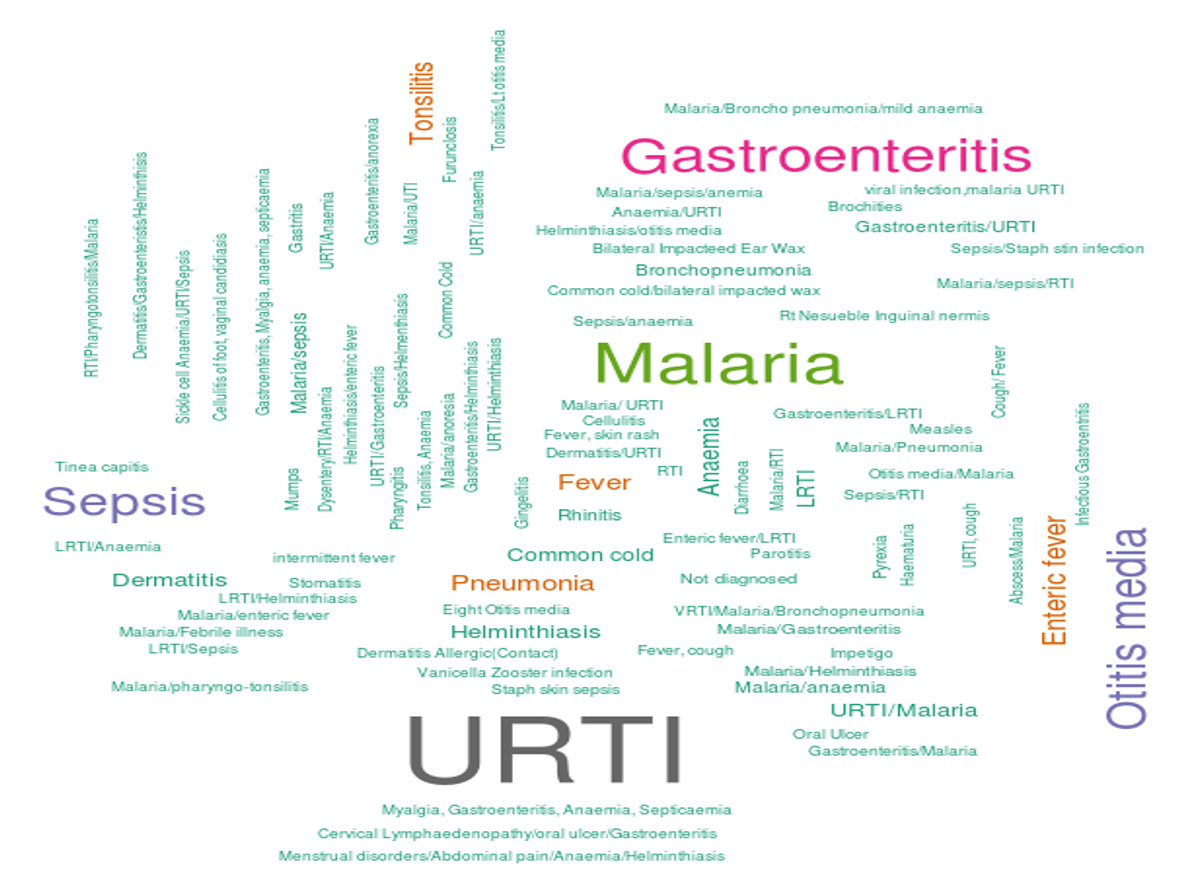
**

Top 150 infections that were reported; with majority of the people having upper respiratory tract infections (URTI), followed by Malaria, Gastroenteritis, Sepsis, Otitis media, and Fever. The rest of the diagnosis had a frequency less than 2.

## Fig. S2. Artificial Neural Network Schematic.


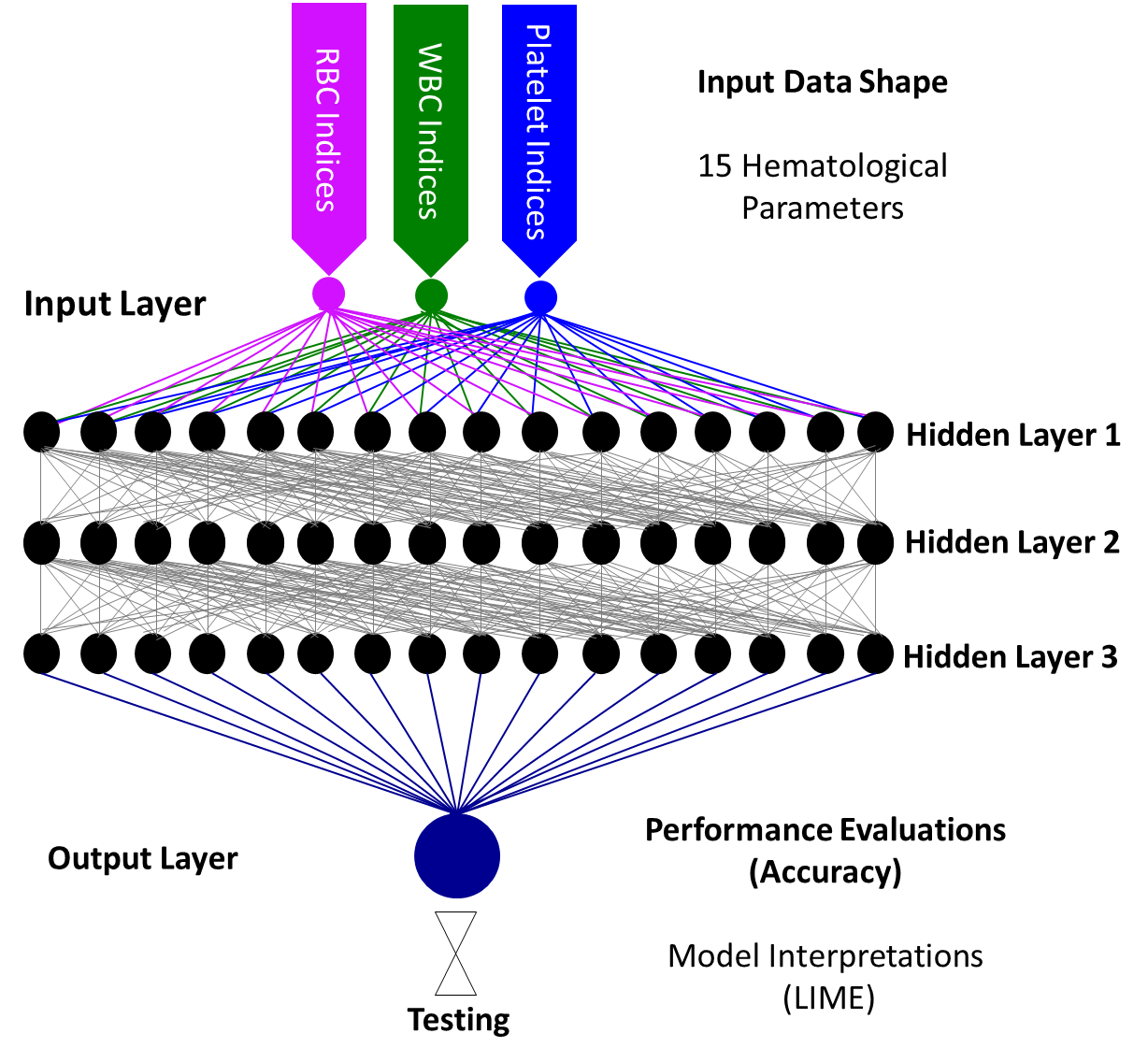


A representative Keras model that is composed of a linear stack of input layers, three hidden layers and one output layer. The input layer composed of an input shape of 15 haematological parameters (RBC parameters, WBC parameters, and Platelet indices). The hidden layers are each composed of a represetative16-stacked unit with ReLU as the activation function. The output layer had sigmoid/softmax function as the activation function and uniform initialization. Interpretations of the model classifications were made using local interpretable model-agonistic explanations (LIME Package in R) [41].

## Fig. S3. Plot for the training and validation history of the ANN.

**
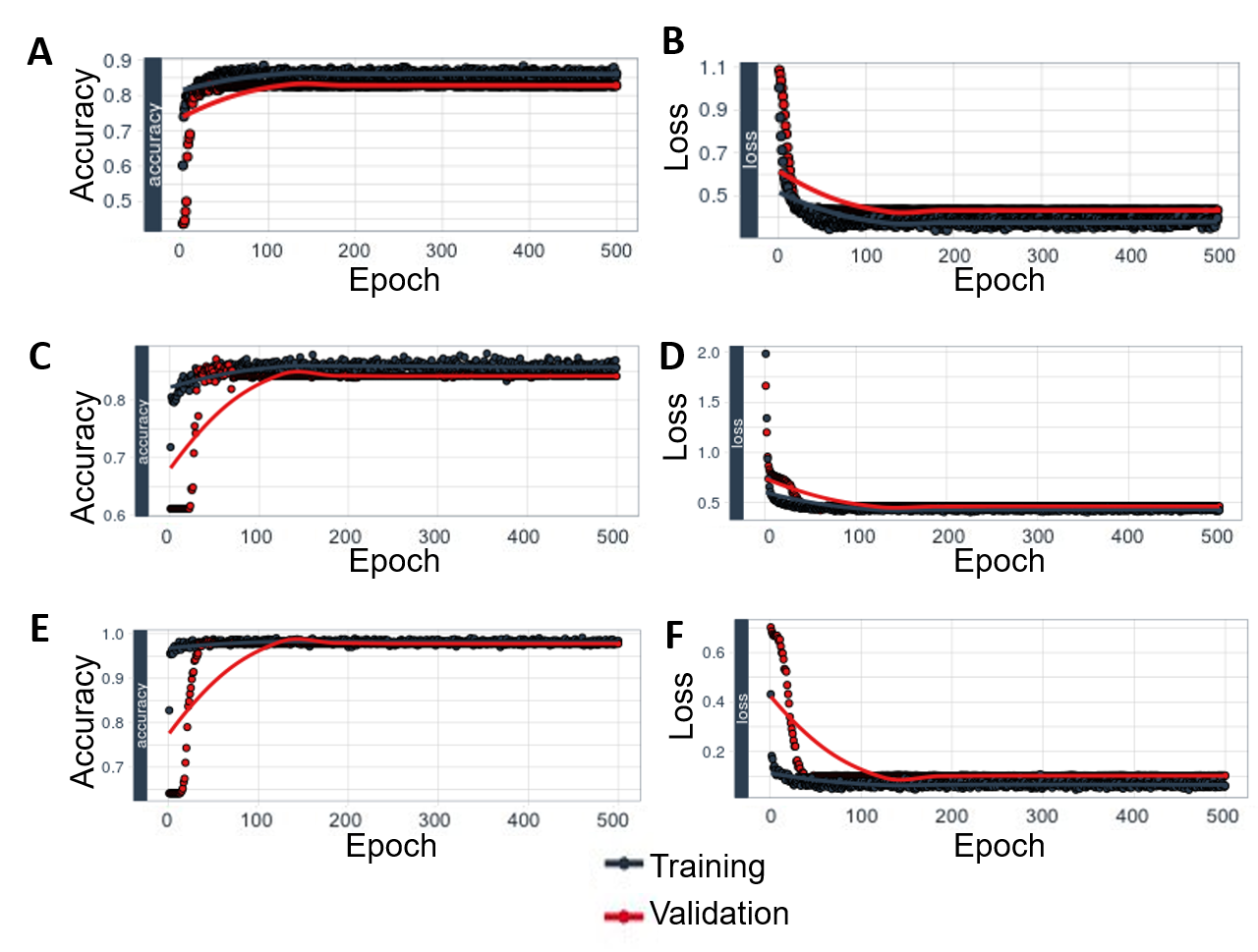
** The figure indicates training and validation history of the model, which shows how accuracy and loss are leveling off, as well as the divergence between training and validation accuracy and training and validation loss. (A and B) Multi-classification of (SM vs. UM vs. nMI) accuracy and loss respectively. (C and D) Accuracy and loss respectively for ANN (UM vs. nMI) binary classifier. (E and F) Accuracy and loss respectively for the ANN (SM vs. nMI) binary classifier. The plots show a minimal model gap between training and validation.

## Fig. S4. Case by case analysis of the classification capability of the ML models.


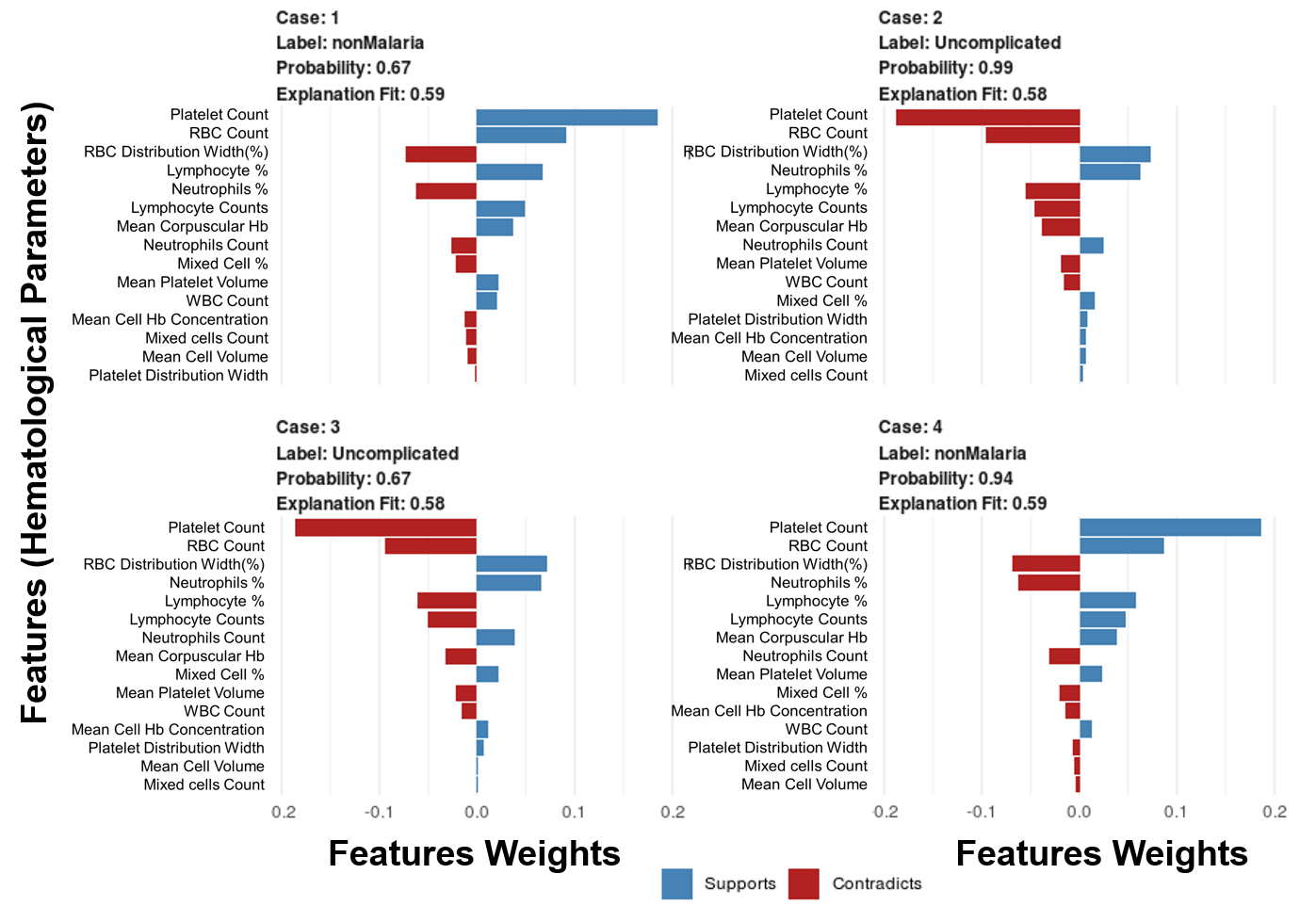


Samples of four cases in the test dataset were selected to indicate the predictions for each case. Case 1 and 4 are nMI patients, while case 2 and 3 are UM patients. The bars indicate the feature weights for each haematological parameter and whether it is a classifier of malaria (supports) or not (contradicts). This figure highlights how the parameters can be used for precision medicine.

## Fig. S5. Density estimates of the haematological parameters between nMI and UM cases for sub-sampled data from Kintampo only.


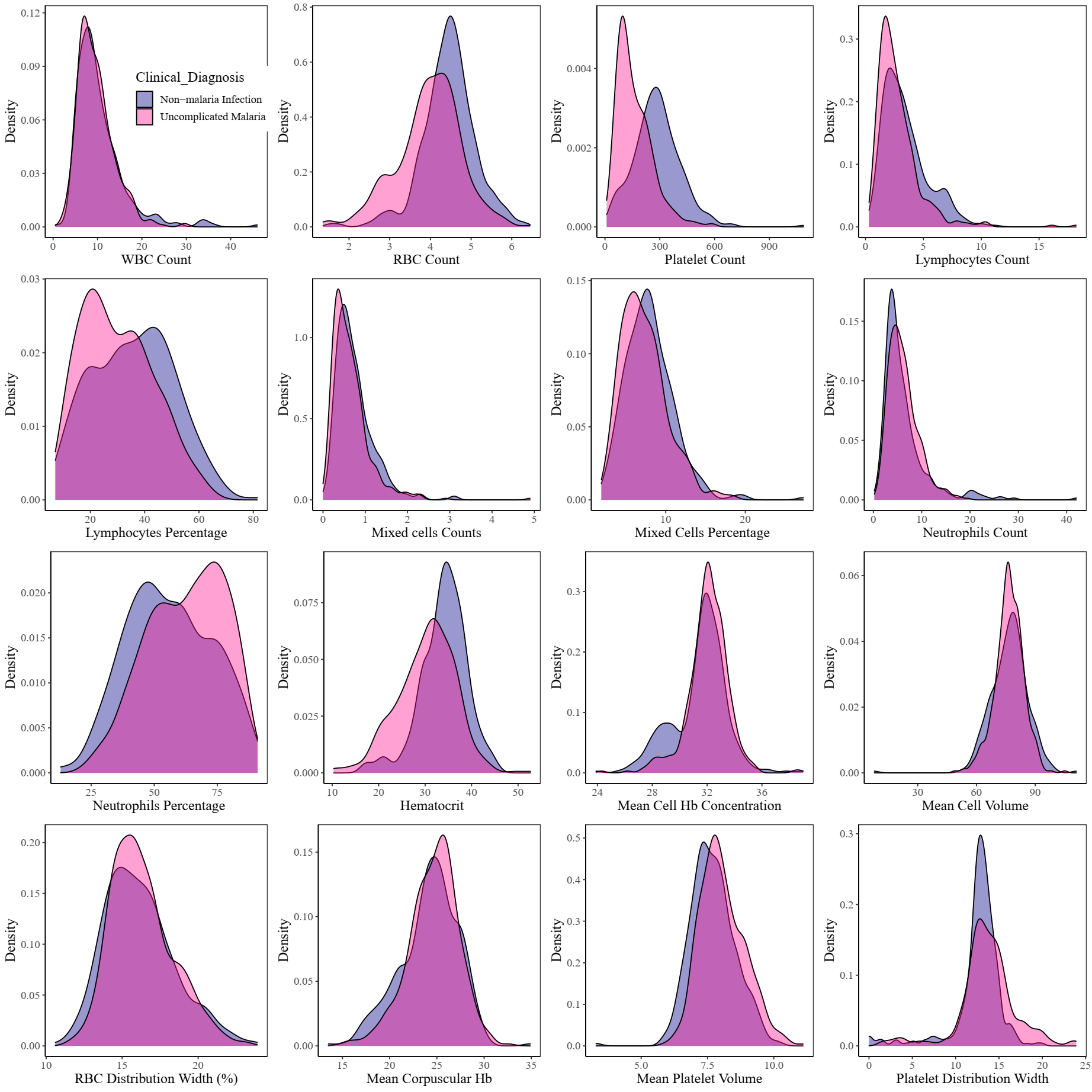


## Fig. S6. Density estimates of the haematological parameters between nMI, and UM cases for sub-sampled data from Kintampo only, as well limit of children under 4 years of age.


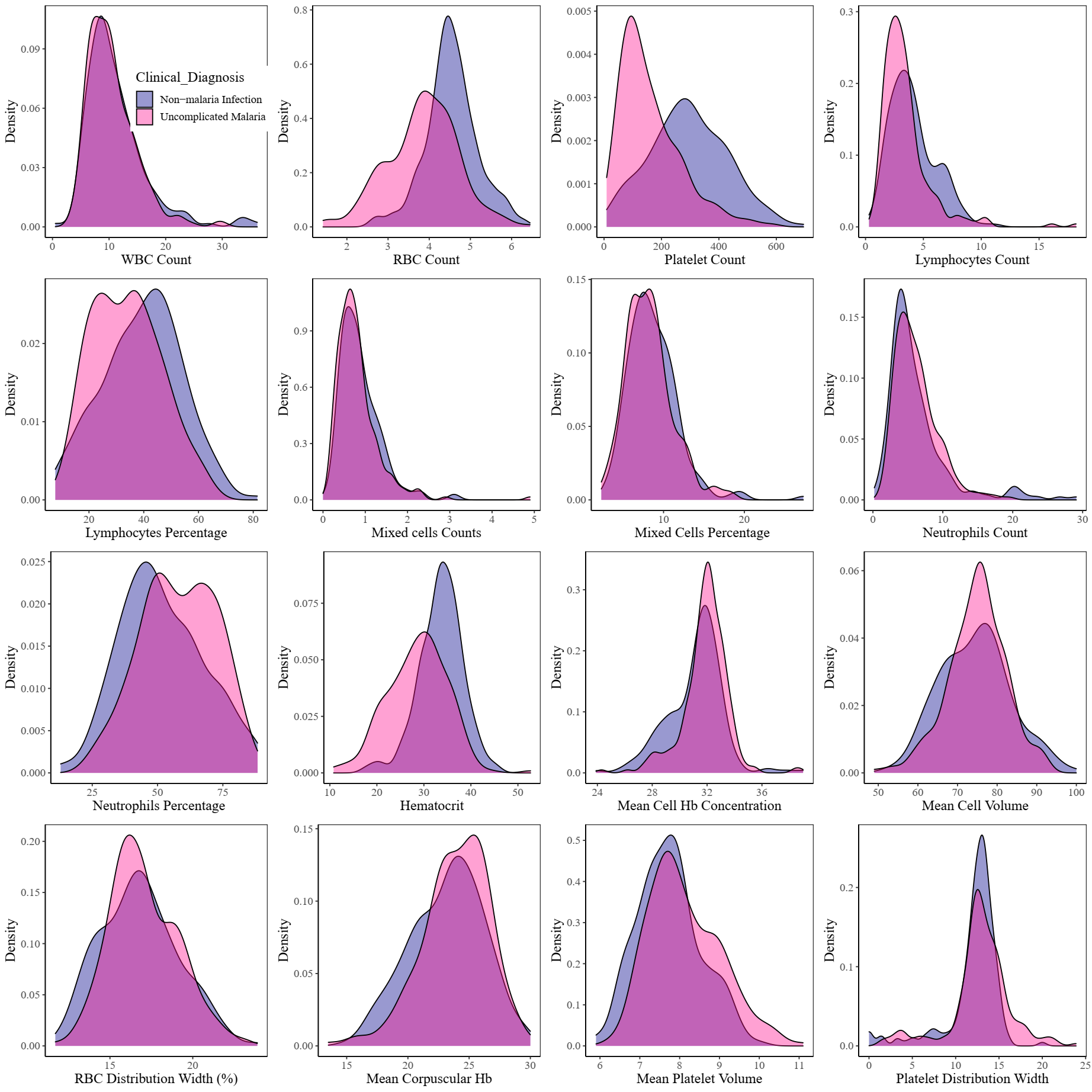

Supplement: Supplementary file 1 — Additional file 1: Table S1. The list of haematological parameters adopted from laboratory procedure manual by the CDC [50]. Table S4. The odds ratio of median categories providing the odd of being diagnosed with either nMI, UM, and SM. The median categories were; low and high levels. Table S5. Performance evaluation of six machine learning models to classify clinical malaria outcomes. Fig. S1. Word cloud of clinical manifestations using clinicians/doctors notes or suspected infections. Fig. S2. Artificial Neural Network Schematic. Fig. S3. Plot for the training and validation history of the ANN. Fig. S4. Case by case analysis of the classification capability of the ML models. Fig. S5. Density estimates of the haematological parameters between nMI and UM cases for sub-sampled data from Kintampo only. Fig. S6. Density estimates of the haematological parameters between nMI, and UM cases for sub-sampled data from Kintampo only, as well limit of children under 4 years of age. [file 12916_2020_1823_MOESM1_ESM.docx]
